# Chidamide (a Histone Deacetylase Inhibitor) Plus Abiraterone in Metastatic Castration‐Resistant Prostate Cancer (mCRPC): A Phase Ib Trial

**DOI:** 10.1002/mco2.70470

**Published:** 2025-11-05

**Authors:** Qi‐xiang Rong, Mei‐ting Chen, Wei Yang, Ri‐qing Huang, Di‐tian Shu, Yue Zhang, Cong Xue, Yu‐chen Cai, Xin An, Hai‐feng Li, Yan‐xia Shi

**Affiliations:** ^1^ State Key Laboratory of Oncology in South China, Collaborative Innovation Center of Cancer Medicine Sun Yat‐sen University Cancer Center Guangzhou Guangdong China; ^2^ Department of Medical Oncology Sun Yat‐sen University Cancer Center Guangzhou Guangdong China

**Keywords:** clinical trial, epigenetic regulation, histone deacetylase, metastatic castration‐resistant prostate cancer

## Abstract

The prognosis of metastatic castration‐resistant prostate cancer (mCRPC) remained unsatisfactory currently. Chidamide is a well tolerated, selective histone deacetylase (HDAC) inhibitor, but the efficacy in mCRPC remained uncertain. From August 2020 to October 2022, a total of 18 patients were enrolled. The primary endpoint was to assess the safety and the secondary endpoints including efficacy and biomarker analysis. The common adverse events (AEs) included anemia, anorexia, hypoalbuminemia, hyponatremia, nausea and fatigue. Grade 3 toxicities included anemia and thrombocytopenia, and no DLT was observed in this study. The median progression‐free survival (PFS) was 3.7 months (95% CI, 0.922–6.611 months), and the median OS was 11.0 months (95% CI, 2.232–19.768 months). The results of the RNA‐seq profile indicated the high immune cell infiltration and the upregulation of immune cell functions in tumor tissues was associated with the efficacy of chidamide, as revealed by GSEA and ssGSEA. Furthermore, chidamide has been demonstrated to upregulate immune response‐related pathways in CRPC cells. Our study suggested that chidamide plus abiraterone is well tolerated in mCRPC, and preliminary evidence suggests that it may improve the survival of patients with mCRPC. Furthermore, combining chidamide with immunotherapy could be another promising option for further enhancing its efficacy.

## Graphical abstract

Metastatic castration‐resistant prostate cancer (mCRPC) remains challenging. This Phase Ib trial tested chidamide, a selective HDAC inhibitor, plus abiraterone in 18 patients. The regimen was well tolerated, with limited Grade 3 toxicities. Median PFS and OS were 3.7 and 11.0 months. RNA sequencing suggested chidamide's immunomodulatory role, supporting further exploration with immunotherapy.

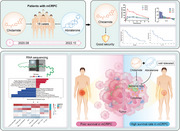

## 1 Introduction

Dysregulated androgen receptor (AR) signaling has been identified as a key driver in prostate cancer development, which has led to the use of androgen deprivation therapy (ADT) as the mainstay treatment for advanced or metastatic disease [1]. However, most patients eventually progress from androgen‐dependent prostate cancer (ADPC) to metastatic castration‐resistant prostate cancer (mCRPC) following initial treatment [2]. The emergence and development of mCRPC involve the regulation of various signaling molecular pathways, with the reactivation of the AR signaling pathway being a key driver. Until now, the first‐line standard treatment for mCRPC remains ADT combined with second‐generation hormone therapies such as abiraterone and enzalutamide, or chemotherapy such as docetaxel and cabazitaxel [3, 4]. For patients progressing after the aforementioned treatments, the use of Sipuleucel‐T is recommended [5]. However, the survival outcomes after second‐line treatment are generally poor in mCRPC. The median overall survival (mOS) after progressing on first‐line treatment is approximately 9–13 months [6]. For patients receiving second‐line and subsequent treatments, the median progression‐free survival (mPFS) typically ranges from about 3 to 5 months [7]. Given these challenges, exploring new treatment strategies for this subset of patients remains a critical priority.

Epigenetic alterations play a significant role in the initiation and progression of cancer [8]. Unlike genetic mutations, which involve changes to the DNA sequence, epigenetic alterations are reversible and influence gene expression without altering the underlying genetic code. These modifications can activate oncogenes or silence tumor suppressor genes, thereby promoting cancer cell growth, survival, and metastasis [9]. In prostate cancer, aberrant DNA methylation patterns have been associated with disease aggressiveness and poor prognosis, while deregulated noncoding RNAs, such as microRNAs and long noncoding RNAs, have been shown to modulate AR signaling and other oncogenic pathways [10, 11]. Thus, targeting epigenetic alterations provides a promising strategy to overcome therapeutic resistance and develop novel treatment approaches for mCRPC.

Histone deacetylases (HDACs) are enzymes that play a pivotal role in the regulation of gene expression by mediate the post‐translational acetylation of various histine proteins. Previous researches have shown that the overexpression of HDACs was related to poor survival in several kinds of tumors [12]. Notably, HDAC1 and HDAC2 are associated with Gleason grade and recurrence‐free survival (RFS) in prostate cancer [13]. Several HDAC inhibitors have been demonstrated therapeutic efficacy by clinical trials, including vorinostat (SAHA) in cutaneous T‐cell lymphoma (CTCL) [14], panobinostat in multiple myeloma [15], romidepsin in peripheral T‐cell lymphoma (PTCL) [16], and chidamide in PTCL, breast cancer and colorectal cancer [17, 18, 19]. Chidamide is a selective inhibitor of HDAC 1, 2, 3, and 10, which has been demonstrated its efficacy in clinical studies across various tumor types [20]. However, its efficacy in mCRPC has only been validated at the preclinical stage.

Therefore, we initiated a Phase Ib clinical trial of the combination of chidamide and abiraterone in patients with mCRPC to evaluate its safety, tolerability, and effectiveness. At the same time, based on the molecular characteristics of the enrolled patients, we aim to predict which patients would benefit from chidamide and identify potential future treatment strategies.

## 2 Results

### 2.1 Patient Characteristics

**FIGURE 1:**
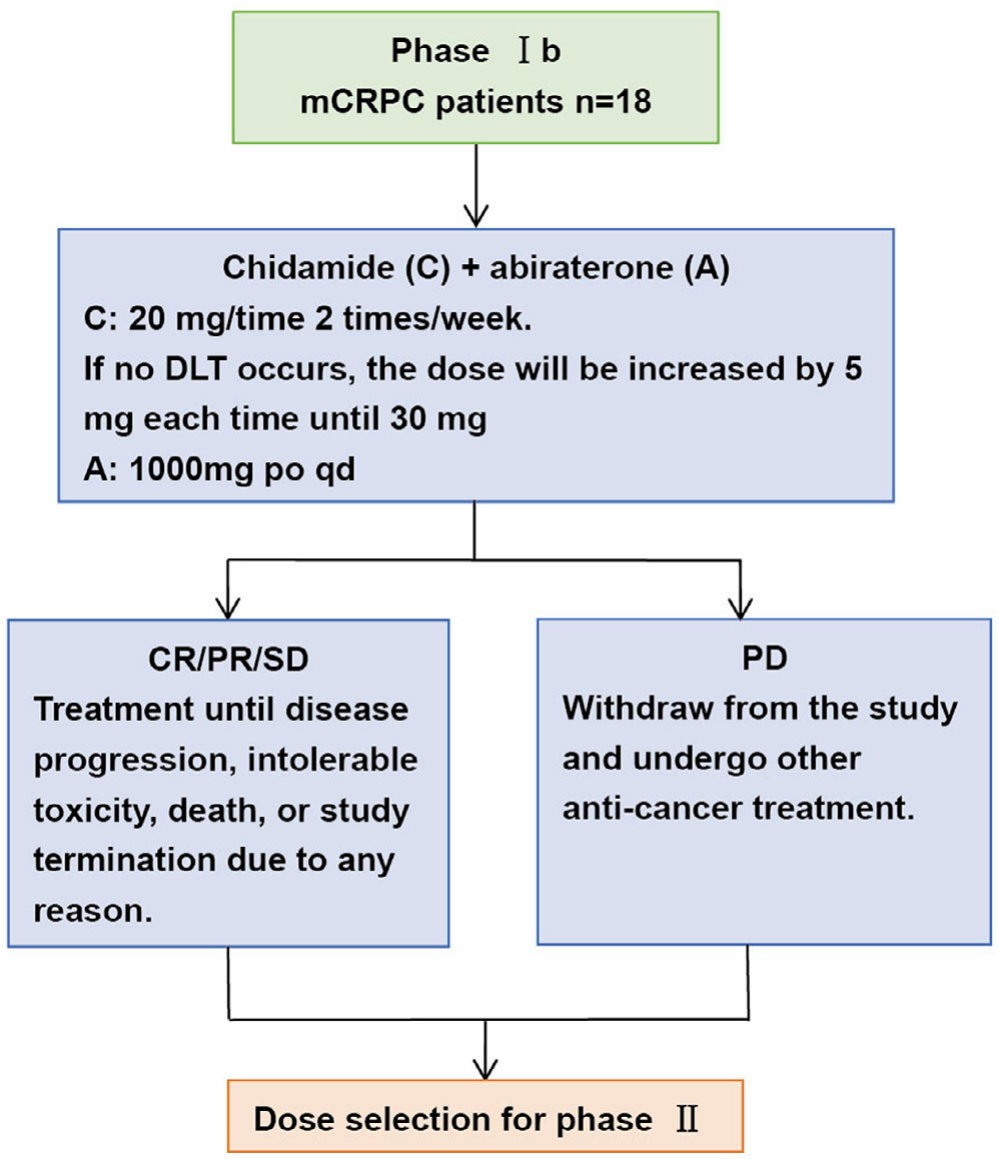
Flow chart of the study patients with metastatic castration‐resistant prostate cancer. CR, complete response; mCRPC, metastatic castration‐resistant prostate cancer; PD, progressive disease; PR, partial response; SD, stable disease.

At the time of data cut‐off was March 08, 2024, a total of 18 male patients (median age, 66.3 years) were enrolled from August 2020 to October 2022, with details of patient recruitment presented in Figure 1. Most common sites of metastases were bone (83.3%) and lymph nodes (44.4%), while 27.8% of patients had visceral metastases. Sixteen patients (88.9%) had progressed on prior abiraterone, of whom four had also received prior enzalutamide. And one patient (5.6%) had progressed on prior enzalutamide. Fourteen patients (77.8%) received docetaxel treatment before enrollment. Six patients (33.3%) presented with visceral metastasis, nine patients (50.0%) presented with bone metastasis and three patients (16.7%) presented with only lymphnode metastasis. Baseline characteristics of the patients are listed in Table 1.

**TABLE 1:** Clinical characteristics of patients treated with chidamide plus abiraterone.

	20 mg (*n* = 4)		25 mg (*n* =11)		30 mg (*n* = 3)	
	No.	%	No.	%	No.	%
Median age, years		69		64		67
Gleason score						
7	1	25	2	18	0	0
> 7	3	75	7	64	2	67
Median serum PSA at baseline (ng/mL)		771		181		108
Elevated plasma LDH (range, U/L)	1 (147–305)	25	4 (144–502)	36	2 (242–324)	67
Elevated plasma ALP (range, U/L)	3 (106–158)	75	4 (74–227)	36	0 (79–119)	0
PCWG3 prognostic group, metastases						
Visceral ± other sites of disease	1	25	4	36	1	33
Bone ± lymph node	3	75	4	36	2	67
Lymph node only	0	0	3	27	0	0
Prior docetaxel	3	75	9	82	2	67
Prior enzalutamide	1	25	4	36	0	0
Prior abiraterone	3	75	10	91	3	100
**Prior chemotherapy lines**						
Chemo‐naïve	1	25	2	18	1	33
Therapy line						
Second line	2	50	6	55	1	33
≥ Third line	1	25	3	27	1	33

### 2.2 Safety

All 18 patients received at least one cycle of combination therapy and were evaluable for dose‐limited toxicity (DLT) and safety. The median relative dose intensity was 100% for each dose level. Overall the most common treatment‐related adverse events (TRAEs) of any grade (Table 2) were anemia (88.9%), anorexia (77.8%), hypoalbuminemia (66.7%), hyponatremia (61.1%), nausea (61.1%), fatigue (61.1%), hypokalemia (38.9%), hyperlipidemia (38.9%), leukopenia (27.8%), and thrombocytopenia (27.8%). Grade 3 TRAEs occurred in five patients (27.8%), included anemia (22.2%) and thrombocytopenia (16.7%). And there was no Grade 4 TRAEs occurred.

**TABLE 2:** Treatment‐related adverse events of chidamide plus abiraterone.

	20 mg (*n* = 4)		25 mg (*n* = 11)		30 mg (*n* = 3)		Total (*n* = 18)	
	Grade 1/2, *n* (%)	Grade 3/4, *n* (%)	Grade 1/2, *n* (%)	Grade 3/4, *n* (%)	Grade 1/2, *n* (%)	Grade 3/4, *n* (%)	Any grade, *n* (%)	Grade 3/4, *n* (%)
Anemia	3 (75)	1 (25)	6 (54.5)	3 (27.3)	3 (100)	0	16 (88.9)	4 (22.2)
Leukopenia	0	0	4 (36.4)	0	1 (33.3)	0	5 (27.8)	0
Neutropenia	0	0	4 (36.4)	0	0	0	4 (22.2)	0
Thrombocytopenia	1 (25)	0	4 (36.4)	2 (18.2)	0	1 (33.3)	5 (27.8)	3 (16.7)
Hypoalbuminemia	3 (75)	0	7 (63.6)	0	2 (66.7)	0	12 (66.7)	0
Hyponatremia	4 (100)	0	5 (45.5)	0	2 (66.7)	0	11 (61.1)	0
Hypokalemia	0	0	7 (63.6)	0	0	0	7 (38.9)	0
Hyperkalemia	0	0	0	0	1 (33.3)	0	1 (5.6)	0
Hyperlipidemia	2 (50)	0	3 (27.3)	0	2 (66.7)	0	7 (38.9)	0
Hyperglycemia	2 (50)	0	2 (18.2)	0	0	0	4 (22.2)	0
Aspartate aminotransferase increase	0	0	1 (9.1)	0	0	0	1 (5.6)	0
Anorexia	4 (100)	0	8 (72.7)	0	3 (100)	0	14 (77.8)	0
Nausea	2 (50)	0	6 (54.5)	0	3 (100)	0	11 (61.1)	0
Fatigue	3 (75)	0	6 (54.5)	0	2 (66.7)	0	11 (61.1)	0
Vomiting	2 (50)	0	2 (18.2)	0	0	0	4 (22.2)	0
Diarrhea	0	0	1 (9.1)	0	3 (100)	0	4 (22.2)	0
Constipation	0	0	3 (27.3)	0	0	0	3 (16.7)	0
Dizziness and headache	0	0	1 (9.1)	0	1 (33.3)	0	2 (11.1)	0

During the dose‐escalation phase, none of the patients experienced a DLT during chidamide treatment. However, patients in the 30 mg cohort experienced persistent Grade 1–2 adverse events, including nausea, anorexia, and fatigue. These symptoms, while not meeting the criteria for DLTs, significantly affected treatment compliance, especially given the older age and poor performance status of the enrolled patients. Moreover, efficacy assessment showed no additional clinical benefit in the 30 mg group compared to the 25 mg group. In consideration of the above, the investigators concluded that subsequent enrollment should proceed at the 25 mg dose level.

### 2.3 Serum Pharmacokinetics

**FIGURE 2:**
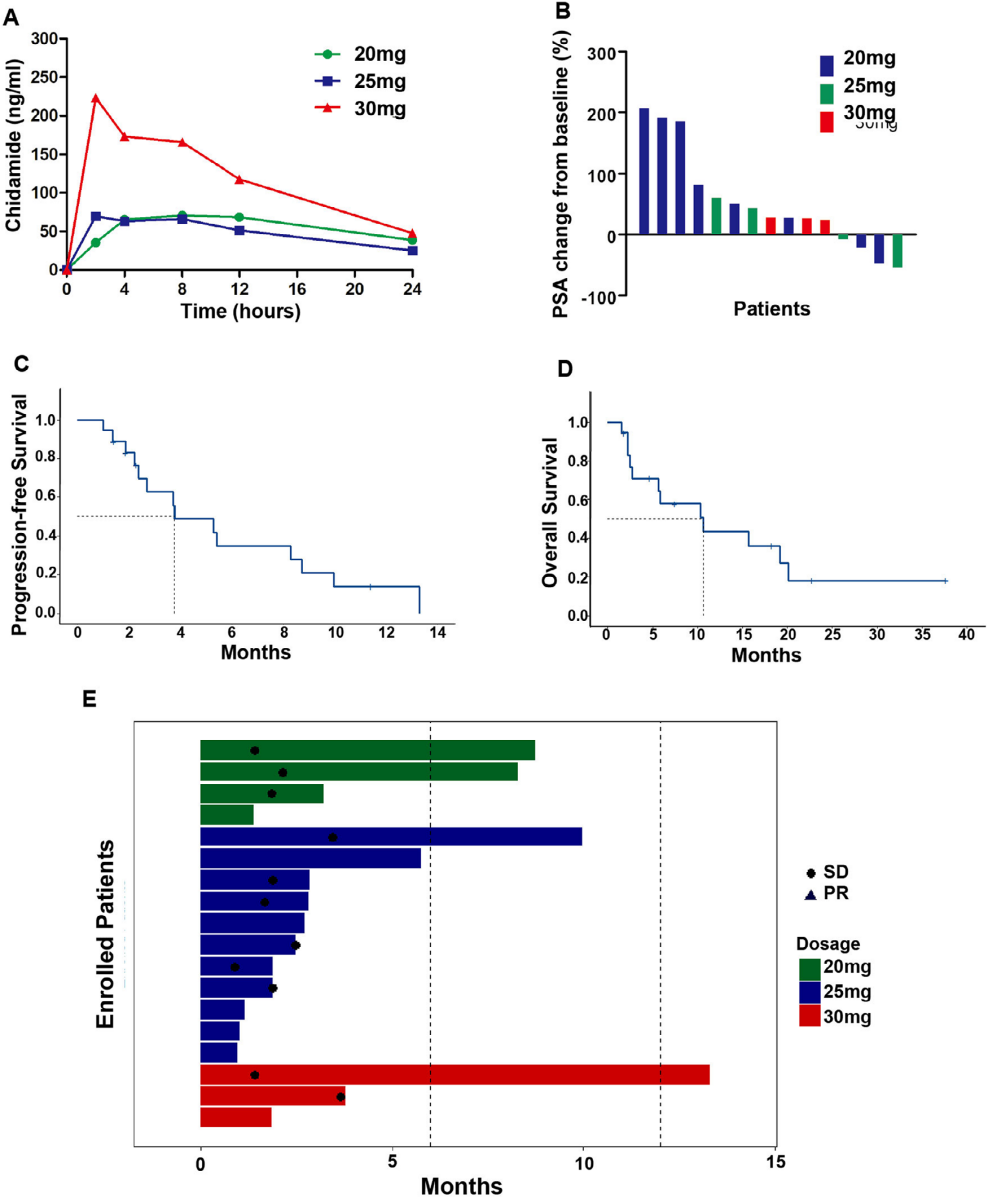
The efficacy of chidamide plus abiraterone in mCRPC patients. (A) Mean plasma concentrations of chidamide at the time points of 2, 4, 8, 12, and 24 h after the completion of the first administration; (B) Best PSA response depicted as the percent change from baseline to the lowest value on treatment below baseline or from baseline to the first recorded; (C) Kaplan–Meier estimates of progression‐free survival (PFS) for patients receiving chidamide plus abiraterone; (D) Kaplan–Meier estimates of overall survival (OS) for patients receiving chidamide plus abiraterone; (E) The PFS of chidamide plus abiraterone for patients in arm 20 mg, arm 25 mg, and arm 30 mg.

The pharmacokinetic (PK) parameters estimated by compartmental modeling using Phoenix Winnonlin 7.0 were summarized in Table 3 [21]. The mean chidamide plasma concentrations versus time curves during cycles 1 at designed time points were shown in Figure 2A. It worth noting that the peak concentration (*C*_max_, 79.189 ± 22.459 vs. 91.155 ± 45.253 vs. 295.900 ± 111.608 ng/mL) and AUC_0–24 h_ (1.331 ± 0.312 vs. 1.157 ± 0.417 vs. 2.860 ± 0.915 h µg/mL) were higher while the volume of distribution at steady state (Vss, 206.877 ± 77.357 vs. 326.340 ± 237.651 vs. 132.045 ± 45.242 L) was lower at the dosage of 30 mg. Systemic clearance and elimination half‐life were comparable among three dosage groups.

**TABLE 3:** Chidamide pharmacokinetic parameter estimates.

	CL (L/h)		Vss (L)		AUC0–24 h (h µg/mL)		*C*_max_ (ng/mL)		*t*_1/2_ (h)	
Dose group	Mean	SD	Mean	SD	Mean	SD	Mean	SD	Mean	SD
20 mg (*N* = 4)	10.218	3.131	206.877	77.357	1.331	0.312	79.189	22.459	13.881	0.993
25 mg (*N* = 11)	15.586	3.592	326.340	237.651	1.157	0.417	91.155	45.253	13.629	6.897
30 mg (*N* = 3)	11.252	3.175	132.045	45.242	2.860	0.915	295.900	111.608	8.062	0.512

### 2.4 Efficacy

With a median follow‐up of 8.0 months, eight patients achieved stable disease (SD), and 10 patients showed progression disease (PD), with a DCR of 44.4%. One patient achieved a decline of PSA over 50.0% (Figure 2B). The median PFS was 3.7 months (95.0% CI, 0.9–6.6 months, Figure 2C), and the median OS was 11.0 months (95% CI, 2.2–19.8 months, Figure 2D). The median PFS of 30 mg group was superior than other groups but failed to meet the statistical boundary (30 mg vs. 25 mg vs. 20 mg = 5.4 vs. 2.7 vs. 3.8 months, Figure 2E). All enrolled patients had experienced either radiographic progression or clinical disease progression.

### 2.5 Biomarker Profiling

In the current genomic analysis, patients with a PFS exceeding 6 months were categorized as good responders, while those with a PFS of less than 6 months were categorized poor responders. For the biomarker studies, we performed RNA‐seq using pretreatment tumor specimens of 3 good responders and 4 poor responders.

**FIGURE 3:**
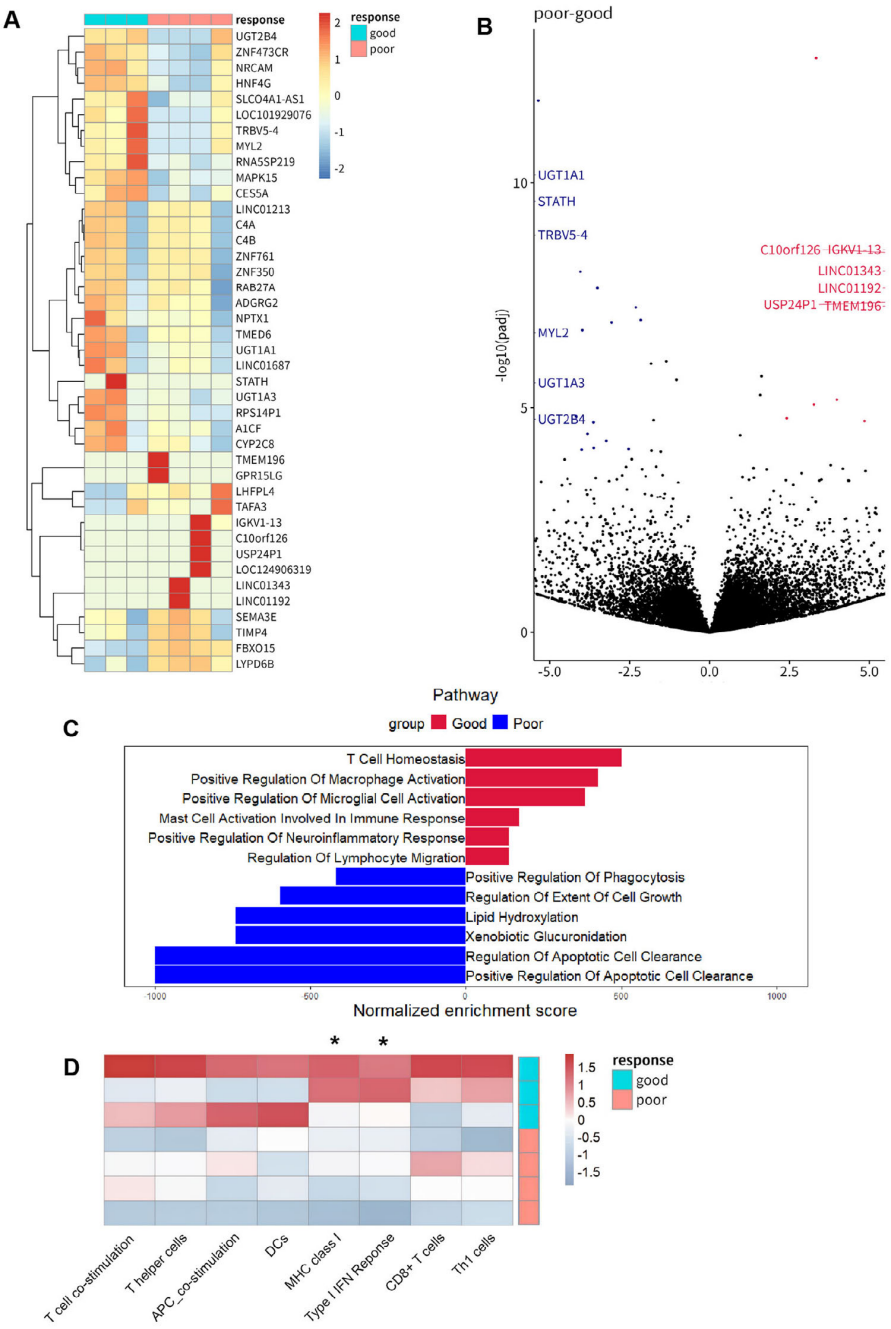
Transcriptome analysis for baseline tumor samples. (A) Heatmap showed the different regulated genes between good responders and poor responders; (B) Volcano map of gene expression of good responders compared to poor responders; (C) GSEA showed the different regulated pathways between good responders and poor responders; (D) The different expression of tumor immune infiltration‐related markers between good responders and poor responders analyzed by ssGSEA.

**FIGURE 4:**
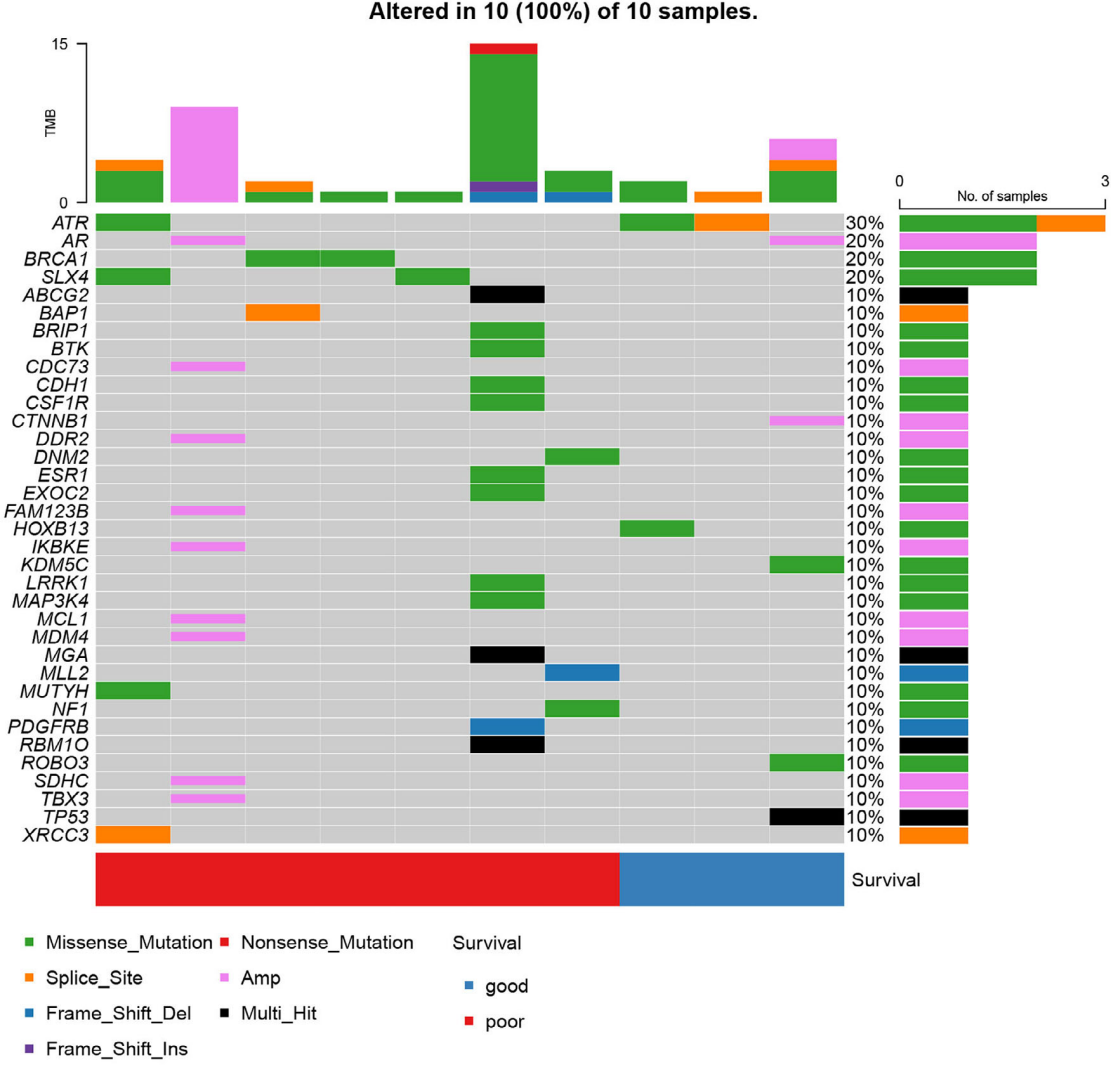
The overview of genomic alterations. (A) The oncoplot of 10 patients in Phase alu

The RNA‐seq profiling found that 27 genes were downregulated and 14 genes were upregulated in good responders compared to poor responders (Figure 3A,B). GSEA analysis indicated that pathways associated with T cell, macrophage, and mast cell functions were enriched in good responders, along with neuroimmune‐related pathways such as microglial activation and neuroinflammatory response pathways (Figure 3C). Further analysis of tumor immune infiltration‐related markers using ssGSEA demonstrated that in the good responders, markers associated with T cells, Th cells, and APC cells were enriched, as well as markers linked to immune response and immune recognition, including MHC‐I and IFN‐α (Figure 3D). Targeted‐sequencing of somatic alteration indicated that BRCA1 had a different alteration trend between two groups (*p* < 0.05, Figure 4A).

**FIGURE 5:**
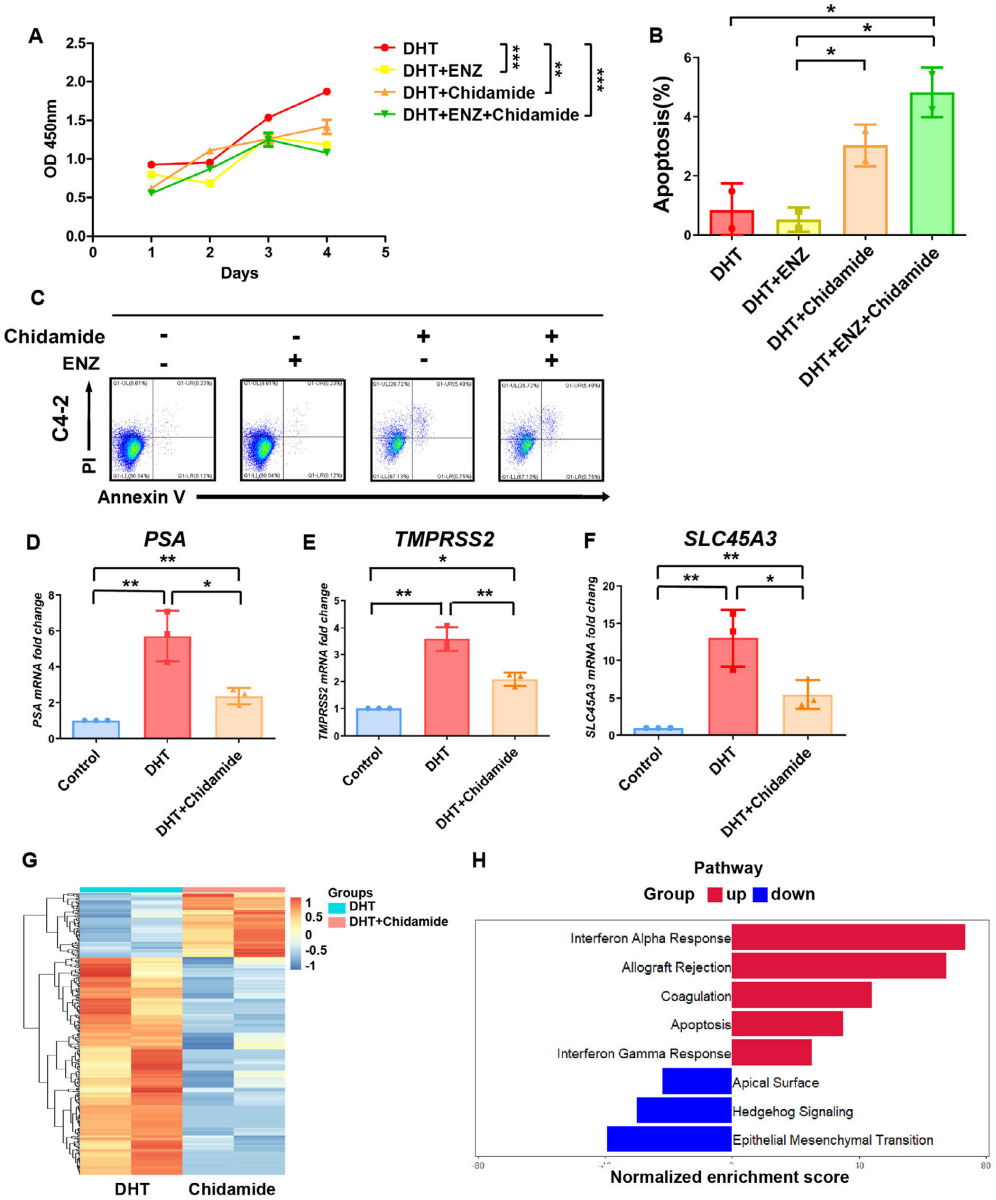
Chidamide inhibited AR pathway and CRPC progression and in vitro. (A) Effect of chidamide and enzalutamide on cell viability. C4‐2 cells were treated with DHT (10 nM), chidamide (0.5 µM), and/or enzalutamide (20 µM) for 96 h. Cell viability was measured by CCK‐8. (B) The percentage of apoptotic C4‐2 cells treated with chidamide and/or enzalutamide, the DHT group was used as the control group for normalization; (C) Scatter plots that represent the percentage of apoptotic C4‐2 cells treated with DHT (10 nM), chidamide (0.5 µM) and/or enzalutamide (20 µM) for 48 h; (D–F) Relative mRNA expression levels of PSA, TMPRSS2, and SCL45A3 were decreased by chidamide (0.5 µM) in C4‐2 cells, the control groups were used for normalization; (G) Heatmap showed the different regulated genes of C4‐2 cells before and after treated by chidamide (0.5 µM). DHT (10 nM) served as control; (H) GSEA showed the different regulated pathways of C4‐2 cells before and after treated by chidamide (0.5 µM). DHT (10 nM) served as control.

In vitro study showed that chidamide effectively inhibits the cell viability of CRPC cells, and further enhanced the inhibition of CRPC cell viability and promotes apoptosis in combination with enzalutamide (Figure 5A, C). The real‐time polymerase chain reaction (RT‐PCR) analysis indicated that chidamide significantly downregulated AR downstream genes, including PSA, TMPRSS2, and SLC45A3 (Figure 5D, F). RNA‐seq showed that 142 genes were downregulated and 29 genes were upregulated in CRPC cells treated with chidamide (Figure 5G). GSEA analysis demonstrated that IFN‐α and IFN‐γ responses, allograft rejection and apoptosis‐related pathways were enriched (Figure 5H).

## 3 Discussion

In the present study, we demonstrated that chidamide plus abiraterone was well tolerated and has certain efficacy in mCRPC patients. Additionally, transcriptome sequencing was conducted on baseline tumor tissues from enrolled patients and cell lines in order to identify biomarkers associated with chidamide treatment and to explore potential future therapeutic strategies. In the Phase Ib study, we achieved a DCR of 44.4% with a median PFS of 3.7 months and tolerable toxicity that was consistent with the results of previous clinical studies of HDAC inhibitors on CRPC (Figure 2C) [22, 23].

The most common AE's included anemia, anorexia, hypoalbuminemia, hyponatremia, nausea, fatigue, hypokalemia, hyperlipidemia, leukopenia, and thrombocytopenia. Grade 3 TRAEs included anemia and thrombocytopenia. No chidamide‐related SAEs were observed. And no unplanned AEs and SAEs were observed. Although no DLTs were observed, the 30 mg dose was associated with a higher frequency of low grade but persistent adverse events that impaired adherence. Furthermore, PK analysis revealed a markedly higher *C*_max_ and AUC at 30 mg compared to 25 mg, which may have contributed to these tolerability issues. Considering safety, tolerability, PKs, and clinical response, we recommend 25 mg as the appropriate dose of chidamide for further evaluation in subsequent clinical trials. In contrast, patients in the 25 mg cohort showed higher CL and Vss, suggesting a faster systemic clearance and wider drug distribution. These PK features may contribute to the better tolerability profile of the 25 mg dose, as well as the higher proportion of patients achieving PSA responses compared to other dose levels (Table 3).

To date, the HDAC inhibitors that have undergone clinical evaluation in mCRPC include romidepsin, entinostat, panobinostat, and chidamide. Romidepsin inhibits HDAC1 and HDAC2, entinostat inhibits HDAC1 and HDAC3, while panobinostat is a pan‐HDAC inhibitor. Chidamide, in contrast, selectively targets HDAC1, HDAC2, HDAC3, and HDAC10, aligning closely with the four HDAC isoforms most overexpressed in prostate cancer, making it a more biologically relevant candidate for mCRPC treatment [24]. The efficacy of HDAC inhibitors in CRPC has been reported in several preclinical and clinical studies. Previous studies have shown that HDAC inhibitors can inhibit CRPC cell growth both in vivo and in vitro, while downregulating AR and AR‐splice variants (AR‐SV) expression and suppressing related downstream pathways [25, 26]. Our study and the study of panobinostat in mCRPC both suggested that HDAC inhibitors could suppress downstream of the AR signaling pathway. Furthermore, in vitro experiments have demonstrated the effectiveness of combining HDAC inhibitors and antiandrogen therapy in inhibiting prostate cancer cell viability, supporting the synergistic interaction between these agents.

In a Phase Ihase en the panobinostat combined with bicalutamide, promising results were shown in CRPC patients who had progressed on the first second‐line antiandrogen treatment. The radiographic progression‐free (rPF) was 1.2 months for 20 mg group and 6 months for 40 mg group [22]. In a Phase Ihase INK ∖ntinostat plus enzalutamide in mCRPC patients who progressed after 1 prior regimen, showed 17.0% of Grade 3 anemia and 4.5 months of median PFS [27]. In the Phase IIhase he K romidepsin in chemotherapy‐naïve mCRPC patients, the time to progression (TTP) was only 1.8 months. The most common Grade 3 adverse event observed with romidepsin and entinostat was anemia, which is consistent with our study [23, 27]. However, in comparison to panobinostat combined with bicalutamide, our study demonstrated a lower incidence of Grade 3–4 TRAEs, particularly thrombocytopenia [22]. In our study, 50% of patients had progressed after two or more second‐line antiandrogen treatments, and 77.8% had progressed after docetaxel, with 27.8% having undergone two or more lines of chemotherapy. Despite these challenging profiles, our study showed a mPFS of 3.7 months and manageable toxicities, indicating that this regimen is a viable option for mCRPC patients.

The expression of HDACs has been shown in numerous studies to be associated with the prognosis of various cancers [12]. Collective data from previous studies and databases show that HDACs are significantly overexpressed in prostate cancer [24]. And the expression of HDACs, particularly HDAC1 and HDAC2, were positively correlated with Gleason scores and shorter PSA relapse time [13]. These findings suggest that HDACs are potential therapeutic targets for multiple cancers, including prostate cancer. Although there was no direct evidence from previous studies, it could be speculated based on past findings that HDACs expression may be related to the efficacy of HDAC inhibitors. Our study lacked the H3 acetylation analysis of patients' baseline tumor tissues, which might explain why some patients achieved longer PFS after chidamide treatment, while others had poorer outcomes.

In the RNA‐seq profiling, we found that the high immune cell infiltration and the upregulation of immune cell functions might be associated with better treatment outcomes. And NGS of a cancer‐related panel of 1021 genes higher immune cell infiltration and upregulation of immune cell functions may be associated with better treatment outcomes. Conversely, patients with poor outcomes demonstrated limited immune activation, indicating that chidamide in combination with immune checkpoint inhibitors (ICIs) may be a promising approach for improving survival. There has growing evidence that HDAC inhibitors can reverse resistance to ICIs [19, 28], which may address the issue of poor efficacy of ICIs in mCRPC [29, 30]. AR was a negative modulator of intratumor CD8^+^ T cells response to ICIs [31]. Increased intratumoral Th1 cells are a key factor for better prognosis in mCRPC treated with ICIs. However, ICIs could not effectively upregulate the proportion of these cells in bone metastatic lesions in mCRPC [32]. This was consistent with our ssGSEA results from RNA‐seq data of baseline tumor tissues. Our research data showed that chidamide could inhibit the AR pathway and upregulate immune response‐related pathways like IFN‐α and IFN‐γ responses related pathways, which may indicate the potential for combining chidamide with ICIs in the treatment of mCRPC. Additionally, NGS analysis revealed that somatic mutation of BRCA1 might be linked to poorer prognosis. This is consistent with previous studies suggesting that BRCA1‐mutated prostate cancers are associated with more aggressive disease [33]. In fact, studies have shown that HDAC inhibitors may enhance the efficacy of other targeted therapies, such as PARP inhibitors or BET inhibitors, particularly in BRCA‐mutated tumors [34, 35, 36, 37]. These findings highlight the potential role of BRCA1 status and immune‐related gene expression profiles as predictive biomarkers to guide patient selection and combination strategies in future trials.

Although the sample size of our study may limit the statistical power and precision of efficacy estimates, this is consistent with the design and objectives of Phase Ihase gh the sample size of our study may limit the statistical power and precision of PK profile of chidamide in combination with abiraterone, as well as to determine the recommended dose for further investigation. Given the encouraging safety profile and preliminary signals of efficacy observed, a subsequent Phase II clinical trial with a larger patient cohort is being planned to further evaluate the therapeutic potential of this combination regimen in mCRPC.

In summary, our study suggests that chidamide plus abiraterone is well tolerated in patients with mCRPC, with preliminary evidence indicating potential survival benefits. Patients with higher baseline expression of immune‐related pathways appeared to derive greater benefit from this regimen, highlighting the potential of biomarker‐guided patient selection. These findings support further clinical evaluation of chidamide‐based combination strategies. We are currently planning Phase II–III trials to investigate the efficacy of combining chidamide with ICIs, aiming to improve outcomes in mCRPC through immune modulation.

## 4 Patients and Methods

### 4.1 Study Design

This was a single‐center, single‐arm, open‐label, dose‐escalation Phase Ib clinical trial to evaluate the PKs, safety, and preliminary efficacy of chidamide plus abiraterone in patients with mCRPC. All cohort data were collected from Sun Yat‐sen University Cancer Center. The primary endpoints included PKs, maximum‐tolerated dose (MTD), DLT, and AEs. The secondary endpoints included efficacy and biomarker analysis. Patients who satisfied the following inclusion criteria were eligible: (1) age ≥ 18 years old, (2) histologic diagnosis of prostate cancer, (3) disease progression after standard treatment (according to the PCWG3 criteria), patients must have failed for at least one type of third‐generation antiandrogen therapy (such as abiraterone or enzalutamide, etc.) and have failed or be unsuitable for docetaxel treatment, considering mCRPC. (4) Eastern Cooperative Oncology Group (ECOG) performance status of 0–1, with an expected survival of longer than 3 months. Prior exposure to HDAC inhibitors was an exclusion criterion for enrollment.

The trial adhered to the principles outlined in the Declaration of Helsinki and the International Conference on Harmonisation Good Clinical Practice guidelines. Ethical approval was granted by the institutional review board of Sun Yat‐Sen University Cancer Center. All participants provided written informed consent prior to enrollment. The study was registered under the clinical trial number ChiCTR2200058569 on www.chictr.org.cn.

### 4.2 Dose Escalation

Chidamide was tested in three dose groups (20 mg, 25 mg, 30 mg). The starting dose was 20 mg per dose, administered twice a week, with a treatment cycle lasting 4 weeks. Dosing occurred on fixed days of each week, with two doses administered on the same day each week for the duration of the 4‐week cycle. Three participants are enrolled in each dosage group. If no DLT occurred during the first treatment cycle, the dose was escalated and three patients were enrolled at the next dose level. However, if one DLT was observed, three additional patients were added at the same dose level for further assessment. If no new DLTs occur, the study continues to the next dose level. If two or more of the six patients at a given dose level developed a DLT, further dose escalation was halted, and the prior dose level was determined to be the MTD. Abiraterone was combined with the second administration of chidamide on Day 4 of the first cycle. The observation for DLT was in the period of first cycle of treatment. Patients who were continuing treatment undergo efficacy evaluations every two cycles.

According to the National Cancer Institute Common Toxicity Criteria (version 5.0), a DLT was defined as any chidamide‐related AE of hematologic Grade 4 persisting for more than 7 days, Grade 3 neutropenia with fever (body temperature of ≥ 38.5°C), Grade 4 thrombocytopenia with bleeding tendency, Grade 4 anemia, or other nonhematologic AE of Grade 3 or 4 persisting for more than 3 days (besides alopecia).

### 4.3 Efficacy Outcome Measures

Disease progression was identified based on the occurrence of one or more of the following criteria: (1) radiographic progression, with or without PSA progression (PSA progression is defined as a relative increase of ≥ 25% and an absolute increase of ≥ 2 ng/mL from the baseline, confirmed 3 weeks later), (2) clinical progression with PSA progression or radiographic progression evaluated using RECIST version 1.1 (clinical progression includes increased pain requiring the use of opioid medication for control and/or worsening of the ECOG performance status to 3 or higher), (3) the decision to initiate a new antitumor treatment, (4) requirement for radiotherapy or surgical intervention due to pathological fractures or spinal cord compression.

### 4.4 Pretreatment and Follow‐Up Evaluations

Eligible patients underwent comprehensive evaluation including medical history, physical examination, laboratory assessments, and imaging studies. Clinical visits were scheduled every 4 weeks, with radiologic evaluations conducted every 8 weeks and upon treatment discontinuation. PSA levels and VAS pain scores were monitored monthly, and all laboratory analyses were performed at a central facility. Survival follow‐up was conducted every 3 months.

### 4.5 Pharmacokinetics Analysis

From patients administered with different doses of chidamide, blood samples were collected in a sodium heparin tube at the time points of 2, 4, 8, 12, and 24 h after the completion of the first administration of chidamide (Day 1) in treatment cycle 1 and cycle 2. PK parameters were estimated from chidamide concentrations measured using serum samples taken at specified time points postdosing, and were analyzed by Phoenix Winnonlin 7.0.

### 4.6 Biomarker Profile

Total RNA was extracted from tumor samples. After quality control of RNA amount, purity, and integrity, cDNA library with 300 ± 50 bp size was generated from ∼ 1 µg of total RNA. Then library was sequenced on an Illumina Novaseq 6000 using 2 × 150 bp paired‐end sequencing chemistry. Differentially expressed genes were defined as fold change > 2 or fold change < 0.5 and *p* < 0.05, and then Gene Ontology (GO) and Kyoto Encyclopedia of Genes and Genomes (KEGG) pathway enrichment analyses were done. All services were provided by LC Biotech Corporation (Hangzhou, China).

### 4.7 Cell Lines and Cell Culture Conditions

C4‐2 cell line was provided by Prof. Yonghong Li (Sun Yat‐sen University Cancer Center) and validated by short tandem repeat (STR). C4‐2 cells were grown in RPMI 1640 containing 10% FBS.

### 4.8 RNA Extraction and Quantitative Real‐Time PCR

Total cellular RNA was isolated using EZ‐press RNA Purification Kit (EZBioscience, USA) according to the manufacturer's protocol. For first‐strand cDNA synthesis, 5 µg of total RNA was reverse‐transcribed using the PrimeScript RT Master Mix (Perfect Real Time, TaKaRa Bio, Kusatsu, Japan) followed by quantitative PCR (qPCR) with 2 × Taq Master Mix (Vazyme Biotech Co., Ltd, China) according to the manufacturer's instructions. Real‐time PCR analyses were conducted using the Biorad CFX96 system and the appropriate primers to estimate the mRNA expression. The primer sequences were as follows: for PSA, forward 5′‐ACGCTGGACAGGGGGCAAAAG‐3′ and reverse 5′‐GGGCAGGGCACATGGTTCACT‐3′; for TMPRSS2, forward 5′‐CAGGAGTGTACGGGAATGTGATGGT‐3′ and reverse 5′‐GATTAGCCGTCTGCCCTCATTTGT‐3′; for SLC45A3, forward 5′‐TCGTGGGCGAGGGGCTGTA‐3′ and reverse 5′‐CATCCGAACGCCTTCATCATAGTGT‐3′; for GAPDH, forward 5′‐CCATCACCATCTTCCAGGAGCGA‐3′ and reverse 5′‐GGTGGTGAAGACGCCAGTGGA‐3′.

### 4.9 Flow Cytometry

Apoptosis was evaluated by flow cytometry using an Annexin V‐fluorescein isothiocyanate (FITC) apoptosis detection kit (Vazyme Biotech Co., Ltd, China) according to the manufacturer's instructions with Annexin V‐FITC staining with propidium iodide (PI), and then analyzed on the CytoFLEX LX Flow Cytometer. Data were analyzed using Beckman CytExpert Software.

### 4.10 Next‐Generation Sequencing

Formalin‐fixed paraffin‐embedded (FFPE) tumor samples and matched peripheral blood or buccal swabs samples were used to extract DNA and analyzed via next‐generation sequencing (NGS) of a cancer‐related panel of 1021 genes (GenePlus [China]).

### 4.11 Statistical Analysis

Statistical analysis was carried out using IBM SPSS statistics software or GraphPad Prism using Student's *t*‐test or one‐way ANOVA or Dunnett's test. All experiments were repeated in triplicate. Data are expressed as mean ± standard deviation (SD). Statistical significance was defined as *p* < 0.05.

## Author Contributions

Yan‐xia Shi had full access to all the data in the study and takes responsibility for the integrity of the data and the accuracy of the data analysis. Study concept and design: Yan‐xia Shi, Xin An, and Hai‐feng Li. Acquisition of data: Qi‐xiang Rong, Mei‐ting Chen, and Wei Yang. Analysis and interpretation of data: Qi‐xiang Rong, Mei‐ting Chen, and Wei Yang. Drafting of the manuscript: Qi‐xiang Rong. Critical revision of the manuscript for important intellectual content: Yan‐xia Shi, Hai‐feng Li, and Xin An. Statistical analysis: Qi‐xiang Rong, Mei‐ting Chen, Ri‐qing Huang, and Hai‐feng Li. Obtaining funding: Yan‐xia Shi and Mei‐ting Chen. Administrative, technical, or material support: Ri‐qing Huang, Di‐tian Shu, Yue Zhang, Cong Xue, and Yu‐chen Cai. Supervision: Yan‐xia Shi, Hai‐feng Li, and Xin An. Other: None. All the authors have read and approved the final manuscript.

## Funding

This work was supported by the grants from National Natural Science Foundation of China (No. 82473400, No. 82403969) and National Key Research and Development Program of China (No. 2021YFE0206300).

## Ethics Statement

This clinical study was approved by the Ethics Committee of Sun Yat‐sen University Cancer Center (Approval No. B2020‐044). All procedures performed in this study involving human participants were in accordance with the ethical standards of the institutional research committee and with the Helsinki Declaration. Written informed consent was obtained from all participants prior to their enrollment in the study.

## Conflicts of Interest

The authors declare no conflicts of interest.

## Data Availability

The data collected and used in this study are available from the corresponding author upon reasonable request. The authenticity of this article has been validated by uploading key raw data to the Research Data Deposit public platform (www.researchdata.org.cn).
