# Correction to “Chidamide (a Histone Deacetylase Inhibitor) Plus Abiraterone in Metastatic Castration‐Resistant Prostate Cancer (mCRPC): A Phase Ib Trial”

**DOI:** 10.1002/mco2.71023

**Published:** 2026-11-01

Q. Rong, M. Chen, W. Yang, et al. “Chidamide (a Histone Deacetylase Inhibitor) Plus Abiraterone in Metastatic Castration‐Resistant Prostate Cancer (mCRPC): A Phase Ib Trial,” *Medcomm* 6, no. 11 (2025): e70470 https://doi.org/10.1002/mco2.70470.

In Figure 5C, the representative Annexin V/PI flow cytometry plots for the DHT control group and the DHT + chidamide group were incorrect. During figure assembly, the representative plot for the DHT + ENZ group was mistakenly duplicated in the DHT control position, and the representative plot for the DHT + ENZ + chidamide group was mistakenly duplicated in the DHT + chidamide position. In the corrected Figure 5C shown below, these two duplicated plots have been replaced with the correct representative plots.

The quantitative data and statistical analysis presented in Figure 5B were generated from the correct original datasets and remain unchanged. This correction does not affect the figure legend, the results reported in the text, any other figure panel, or the conclusions of the article.

 
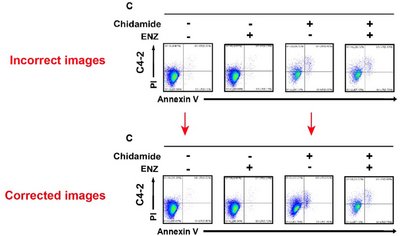


We apologize for this error.

